# Characteristic of extra luminal gastric stromal tumor arising from the lesser curvature of the stomach: A case report

**DOI:** 10.1097/MD.0000000000019885

**Published:** 2020-04-17

**Authors:** Min Wang, Xia Qiu, Xu He, Chuan Tian

**Affiliations:** a Department of Gastroenterology; b Department of Nephrology, The People's Hospital of Nanchuan, Nanchuan District, Chongqing; c Clinical Medical Experimental Teaching Center, School of Clinical Medicine, Chengdu Medical College, District Xindu, Chengdu, Sichuan, China.

**Keywords:** endoscopic ultrasound-guided fine needle aspiration, gastrointestinal stromal tumors, old hemorrhage, pedicle gastric stromal tumor

## Abstract

**Rationale:**

Gastrointestinal stromal tumor (GIST) is one of the most common malignant mesenchymal tumors of the gastrointestinal tract. They generally arise from the fourth layer (muscularis propria) and rarely from the second or third layer. Although the manifestations of gastric stromal tumors are diverse, to our knowledge, there are only several cases of an extra-gastric stromal tumor in the literature appearing with a pedunculation. Pedunculated large GISTs are not frequent and compress the neighboring organs. When they were huge, it is difficult to differentiate the origin of the masses. Thus, in the clinical setting, physicians should pay more attention to the pattern of manifestation of the gastric stromal tumor.

**Patient concerns:**

A 62-year-old man had no gastrointestinal symptoms or significant medical and family histories. During the health examination with US, a cystic-solid tumor was found below liver. The results of the physical examination were unremarkable, and routine laboratory data on admission did not show any abnormal findings.

**Diagnosis:**

Computed tomography of the abdomen showed a mixed echoic mass measuring 10 × 8 × 8 cm and located below the liver, adjacent to the gastric antrum. After endoscopic ultrasound-guided fine-needle aspiration, cytopathology showed that the specimen was filled with red blood cells, and it had no malignant cells. Histopathology revealed that the mass was a GIST, and immunohistochemical analysis showed the following: CD117(+), CD34(+), desmin(−), Dog-1(+), Ki-67% <1%, and smooth muscle actin(−).

**Interventions:**

Surgical resection was performed on the patient.

**Outcomes:**

The lesion was diagnosed as a gastric stromal tumor with a pedicle and an old hemorrhage. The patient's recovery was uneventful. After surgery, computed tomography at the 6-month and 1-year postoperative follow-up visits did not reveal relapse or any metastasis.

**Lessons:**

In the clinical setting, physicians should pay more attention to the pattern of manifestation of the extra-gastric stromal tumor in patients with a pedicle or hemorrhage. Additionally, endoscopic ultrasound-guided fine-needle aspiration can be used to make an accurate preoperative diagnosis of such diseases, and its findings can serve as an important basis for surgical excision of the lesions.

## 1 Introduction

A gastrointestinal stromal tumor (GIST) is one of the most common mesenchymal tumors of the gastrointestinal tract.^[1]^ Most GISTs arise in the stomach and small intestine, and they infrequently arise in other organs.^[2,3]^ Most GISTs are discovered incidentally by gastrointestinal endoscopy. They generally arise from the fourth layer (muscularis propria) and rarely from the second or third layer, and they appear as a round, hypoechoic lesion on endoscopic ultrasonography (EUS). Histologically, they consist of spindle-shaped, epithelial or mixed type cells; more than 95% are c-kit positive (CD117 positive) and 60% to 70% are CD34 positive.^[4–6]^

Extragastric compression lesions often appear similar to gastric submucosal tumors (SMTs) during gastric endoscopy. In those cases, EUS and computed tomography (CT) usually can be used to accurately differentiate extragastric compression from true SMTs.^[7]^ However, in some cases, tumors that did not compress the stomach wall could not be differentiated, even after various methods were used.

Here, we describe a case of specific manifestation of a extragastric stromal tumor with pedicle, old hemorrhage that was misdiagnosed as a cystic-solid lesion below the liver in a patient who had undergone various diagnostic modalities, including endoscopy, EUS, and abdominal CT.

## 2 Case presentation

A 62-year-old man was admitted to the People's Hospital of Nanchuan, and during the health examination with US, a cystic-solid tumor was found below liver. He had no gastrointestinal symptoms or significant medical and family histories (eg, hypertension, pancreatitis, or clinical signs of gallbladder stones). The results of the physical examination were unremarkable, and routine laboratory data on admission did not show any abnormal findings.

**Figure 1:**
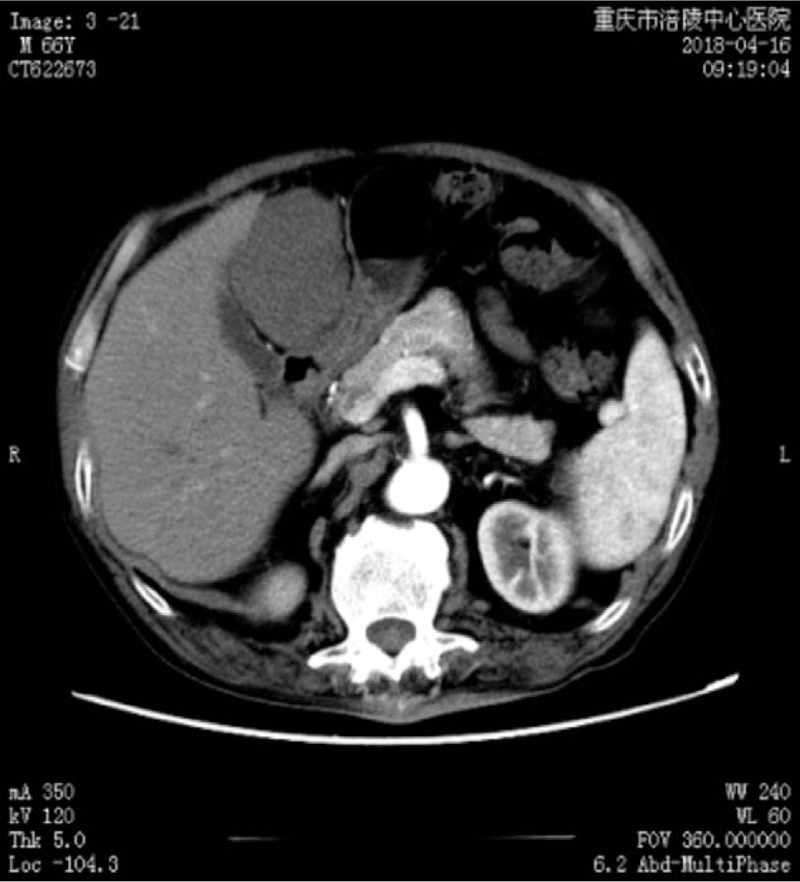
Computed tomography scan of the abdomen. The mixed echoic mass measures 10 × 8 × 8 cm and is located below the liver, adjacent to the gastric antrum. There is no calcification in the mass, and no contrast after injection of a contrast medium.

CT of the abdomen showed a mixed echoic mass measuring 10 × 8 × 8 cm and located below the liver, adjacent to the gastric antrum (Fig. 1). The margins of the mass could not be clearly discriminated from the liver and gastric antrum. There was no calcification of the mass, which showed no contrast after a contrast medium was injected. Radiologically, the mass appeared to originate from the liver and was diagnosed as a benign tumor.

**Figure 2:**
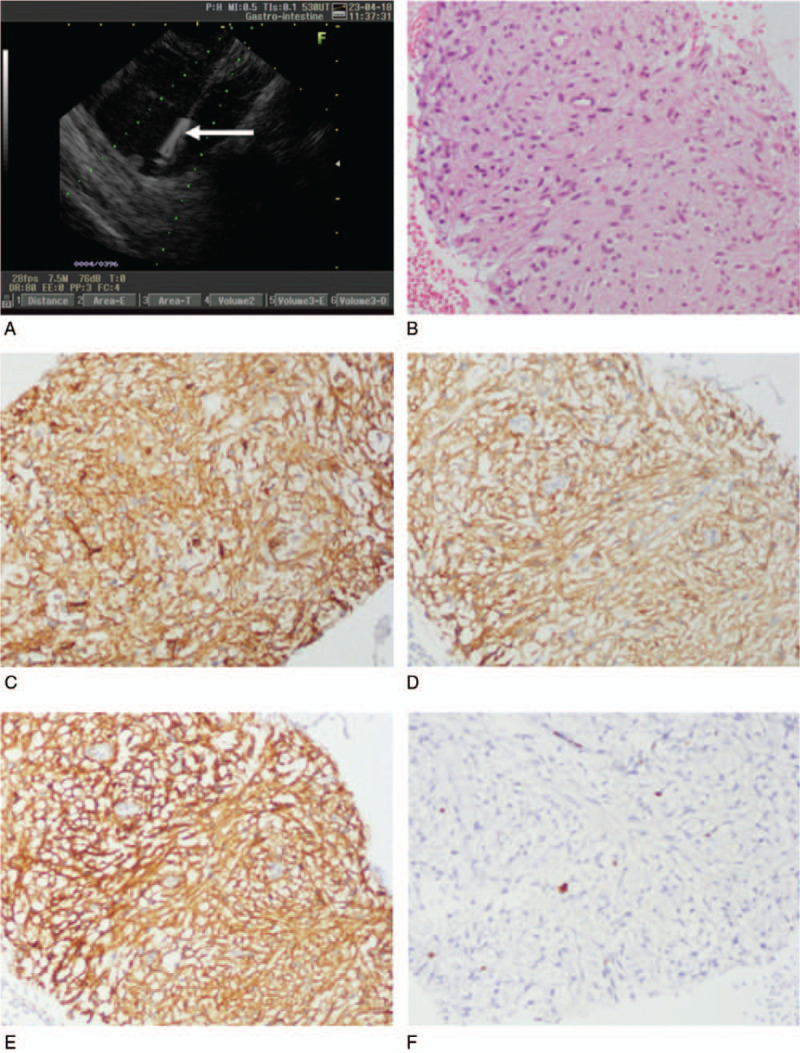
Histopathological and immunohistochemical findings. (A) Puncture of the gastric stromal tumor under ultrasound guidance. Arrow: tip of the needle. (B) Histopathological findings show that the tumor is composed of spindle-shaped cells (hematoxylin-eosin stain, 200×). Immunostaining reveals that (C) the cells stained are positive for CD34 (200×), (D) positive for CD117 (200×), (E) positive for Dog-1 (200×), and positive for Ki <1%.

**Figure 3:**
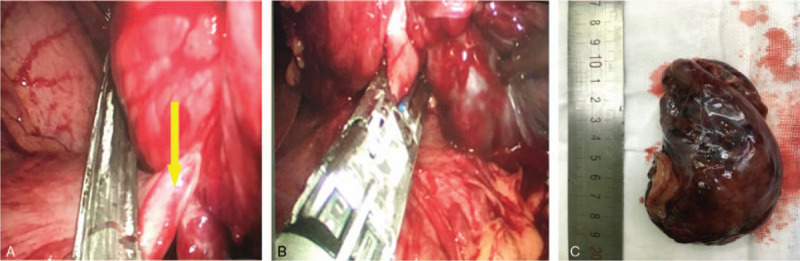
Operative procedure for laparoscopic resection. (A) Laparoscopic view: a cystic-solid mass with a sub-peduncle (yellow arrow) is arising from the lesser curvature of the gastric antrum. (B) Laparoscopic resection of the mass. (C) Postoperative specimen: the diameter of the peduncle is about 3 cm.

Gastric endoscopy showed no abnormal finding in the upper gastrointestinal tract. During the linear EUS examination, there was a bigger cystic-solid mass, mainly cystic, located by the liver and lesser curvature of the gastric body. There was no septations in the mass near the liver, and it was partly solid. The margin of the mass was clear, and no blood flow was detected with color Doppler ultrasonography. Endoscopic ultrasound-guided fine-needle aspiration (EUS-FNA) was performed. After puncturing the cystic lesion, the stylet was removed and 5-mL of negative pressure suction was applied within the lesion. The syringe was filled with an incoagulable bloody fluid. After EUS-FNA, cytopathology showed that the specimen was filled with red blood cells, and it had no malignant cells. However, histopathology revealed that the mass was a GIST, and immunohistochemical analysis showed the following: CD117(+), CD34(+), desmin(−), Dog-1(+), Ki-67% <1%, and smooth muscle actin(−) (Fig. 2A–C). A laparoscopic examination (Fig. 3A–E) was performed for the mass that measured about 10 cm in diameter. A cystic-solid mass with a sub-peduncle arising from the lesser curvature of the gastric antrum was indicated, and it had no involvement with the liver, gallbladder, and small intestine. Postoperatively, we found that the mass was filled with a thick hematoma that was not clotted. The entire mass was sectioned for examination. Histopathologically, the mass was diagnosed as a gastric stromal tumor and categorized in the moderate risk group (mitosis, ≤5/50 high power field; mass diameter, 5.1 × 10.0 cm). Immunohistochemical analysis showed the following: CD117(+), CD34(+), Dog-1(+), and Ki-67% <5%. Additionally, the surgical margin was not involved.

The patient's recovery was uneventful. He was discharged from the hospital on postoperative day 5. After surgery, CT at the 6-month and 1-year postoperative follow-up visits did not reveal relapse or any metastasis. However, we will continue follow-up of this patient.

## 3 Discussion

Most gastric stromal tumors appear as a protruding mass on endoscopy. However, occasionally, extragastric compression may be misdiagnosed as a GIST or SMT by endoscopic examination, and even by EUS. CT is the preferred imaging modality for intraperitoneal masses. It is a helpful method for locating and qualitatively diagnosing GISTs. Nevertheless, EUS-FNA is recognized as an effective technique for obtaining tissue samples, and it is the only accurate modality for diagnosing GIST in real time with minimal risk.^[8–11]^

According to the relationship between the location of the GIST and gastric wall, Kim classified GISTs into 4 types.^[12]^ Among the types, type III is located in the middle of the gastric wall, and type IV protrudes mainly into the serosal side of the gastric wall. If a gastric stromal tumor is a growth in the extra-gastric cavity located close to other organs in the abdominal cavity or if it even compresses the adjacent organs and there are no clinical symptoms, it is easy to misdiagnose as the abdominal cavity or as another organ occupying lesions. According to the best of authors knowledge there were only several published cases reports describes the pedunculated GISTs mimicked hepatic tumor and pancreatic tumor^[13–15]^. Sometimes, the extra gastrointestinal GIST involving lesser omentum, it is also difficult to differentiate the origin, and may be misdiagnosed as hepatic hemangioma and extramucosal tumor of the stomach.^[16–18]^ In our case, however, the gastric stromal tumor was an extra-gastric cavity growth with hematocele that appeared as a cyst, without calcification and another solid mass, adjacent to the liver and porta hepatis. Thus, it was misdiagnosed as an abdominal lesion based on ultrasonography and CT findings. Even findings from the EUS examination did not indicate that the lesion originated from the gastric wall.

Intraoperative laparoscopy showed that the lesion was a pedicle lesion (about 3 cm) with an intact capsule, and it was completely free in the abdominal cavity without an obvious connection to the muscularis propria layer of the gastric wall. After laparoscopic exploration, the lesion was determined to have originated from the gastric wall and be an extra-gastric, pedicellated growth.

Generally, extra-gastrointestinal stromal tumor is not involved the gastrointestinal tract, and there are few manifestations of gastrointestinal hemorrhage. The tumor is usually large, and because the tumor is relatively fragile and lacks the coverage of the digestive tract wall, some of them may be combined with hemorrhage and necrosis in the tumor body. It is very easy to cause iatrogenic intraperitoneal dissemination due to intraoperative rupture. Therefore, in the process of operation, we should try to avoid too much contact with the tumor and prevent tumor rupture.^[19]^ When there is calcification, bleeding and cyst infection, it is more difficult to make a qualitative diagnosis.^[20]^ Because of the small amount of old hemorrhage in the gastric stromal tumor, the lesion was similar to a cyst detected by CT and ultrasonography, which is rare and also increases the difficulty of using routine diagnostic imaging to make an accurate diagnosis. EUS-FNA can provide important information for defining the nature of cystic and solid lesions located around the upper digestive tract. During EUS-FNA, the fluid in the lesion of the present patient was found to be uncoagulated bloody fluid. Combined with results of the cytological examination, it was further confirmed that the fluid was red blood cells. Pancreatic pseudocyst and simple cyst were excluded by the routine examination of the puncture fluid, biochemical indexes, and levels of blood amylase, lipase, and tumor markers. Further, immunohistochemistry confirmed that the lesion was a gastric stromal tumor, and the value-added index of Ki-67 was about 1%, which was important for the successful implementation of laparoscopic resection of a gastric stromal tumor in this patient.

## 4 Conclusion

Although the manifestations of gastric stromal tumors are diverse, to our knowledge, there are no cases of an extra-gastric stromal tumor in the literature appearing with a pedicle or hemorrhage. Thus, in the clinical setting, physicians should pay more attention to the pattern of manifestation of the gastric stromal tumor. Additionally, EUS-FNA can be used to make an accurate preoperative diagnosis of such diseases, and its findings can serve as an important basis for surgical excision of the lesions.

## Acknowledgments

I would like to express special gratitude to Professor Yong Pang, a general surgeon, for kindly providing me with surgical knowledge and his experience.

## Author contributions

All authors equally contributed to this study by interpreting the data, and drafting and writing this manuscript. MW and CT participated in the patient's care during his hospital course including surgery and endoscopic ultrasound-guided fine-needle aspiration; XH performed the pathological and immunohistochemical studies and helped draft the manuscript. XQ helped draft the manuscript and collect the data, especially for English modification. All authors have read and approved this manuscript for publication.

## References

[1] Akahoshi K Oya M Gastrointestinal stromal tumor of the stomach: how to manage? World J Gastrointest Endosc 2010;2:271–7. 21160626 10.4253/wjge.v2.i8.271 PMC2998840

[2] DeMatteo RP Lewis JJ Leung D Two hundred gastrointestinal stromal tumors: recurrence patterns and prognostic factors for survival. Ann Surg 2000;231:51–8. 10636102 10.1097/00000658-200001000-00008 PMC1420965

[3] Miettinen M Sarlomo-Rikala M Lasota J Gastrointestinal stromal tumours. Ann Chir Gynaecol 1998;87:278–81. 9891765

[4] Stamatakos M Douzinas E Stefanaki C Gastrointestinal stromal tumor. World J Surg Oncol 2009;7:61–9. 19646278 10.1186/1477-7819-7-61 PMC2749031

[5] Bucher P Villiger P Egger JF Management of gastrointestinal stromal tumors: from diagnosis to treatment. Swiss Med Wkly 2004;134:145–53. 15106018 10.4414/smw.2004.10530

[6] Hirota S Isozaki K Moriyama Y Gain-of-function mutations of c-kit in human gastrointestinal stromal tumors. Science 1998;279:577–80. 9438854 10.1126/science.279.5350.577

[7] Park JM Kim J Kim HI Hepatic cyst misdiagnosed as a gastric submucosal tumor: a case report. World J Gastroenterol 2008;14:3092–4. 18494066 10.3748/wjg.14.3092 PMC2712182

[8] Akahoshi K Sumida Y Matsui N Preoperative diagnosis of gastrointestinal stromal tumor by endoscopic ultrasound-guided fine needle aspiration. World J Gastroenterol 2007;13:2077–82. 17465451 10.3748/wjg.v13.i14.2077 PMC4319128

[9] Okubo K Yamao K Nakamura T Endoscopic ultrasound-guided fine-needle aspiration biopsy for the diagnosis of gastrointestinal stromal tumors in the stomach. J Gastroenterol 2004;39:747–53. 15338368 10.1007/s00535-004-1383-0

[10] Chatzipantelis P Salla C Karoumpalis I Endoscopic ultrasound-guided fine needle aspiration biopsy in the diagnosis of gastrointestinal stromal tumors of the stomach. A study of 17 cases. J Gastrointestin Liver Dis 2008;17:15–20. 18392238

[11] Akahoshi K Matsui N Sumida Y Diagnosis of the gastric submucosal tumors by endoscopic ultrasonography-guided fine needle aspiration. Endoscopia Digestiva 2009;21:1709–17.

[12] Kim HH Endoscopic treatment for gastrointestinal stromal tumor: advantages and hurdles. World J Gastrointest Endosc 2015;7:192–205. 25789089 10.4253/wjge.v7.i3.192 PMC4360437

[13] Ninomiya S Hiroishi K Shiromizu A Gastrointestinal stromal tumor of the lesser omentum: a case report and review of the literature. J Surg Case Rep 2019;2:1–4. 10.1093/jscr/rjz035 PMC6371855 30792845

[14] Baskiran A Otan E Aydin C Unexpectedly ease surgery for a worrisome abdominal mass: pedunculated GISTs. Int J Surg Case Rep 2013;4:920–2. 23999120 10.1016/j.ijscr.2013.06.012 PMC3785861

[15] Skandalos IK Hotzoglou NF Matsui KCh Giant extra-gastrointestinal stromal tumor of lesser omentum obscuring the diagnosis of a choloperitoneum. Int J Surg Case Rep 2013;4:818–21. 23959407 10.1016/j.ijscr.2013.07.006 PMC3785897

[16] Ogawa H Gotoh K Yamada T A case of KIT-negative extra-gastrointestinal stromal tumor of the lesser omentum. Case Rep Gastroenterol 2012;6:375–80. 22855655 10.1159/000337908 PMC3398081

[17] Trombatore C Palmucci S Angelico G Extra-gastrointestinal stromal tumor of lesser omentum: a challenging radiological and histological diagnosis. Clin Imaging 2015;39:1123–7. 26271149 10.1016/j.clinimag.2015.07.007

[18] Fukuda H Suwa T Kimura F Gastrointestinal stromal tumor of the lesser omentum: report of a case. Surg Today 2009;31:715–8. 10.1007/s005950170077 11510610

[19] Li J Ye Y Wang J Chinese consensus guidelines for diagnosis and management of gastrointestinal stromal tumor. Chin J Cancer Res 2017;29:281–93. 28947860 10.21147/j.issn.1000-9604.2017.04.01 PMC5592117

[20] Rösch T Kapfer B Will U Accuracy of endoscopic ultrasonography in upper gastrointestinal submucosal lesions: a prospective multicenter study. Scand J Gastroenterol 2002;37:856–62. 12190103

